# Similar white matter changes in schizophrenia and bipolar disorder: A tract-based spatial statistics study

**DOI:** 10.1371/journal.pone.0178089

**Published:** 2017-06-28

**Authors:** Letizia Squarcina, Marcella Bellani, Maria Gloria Rossetti, Cinzia Perlini, Giuseppe Delvecchio, Nicola Dusi, Marco Barillari, Mirella Ruggeri, Carlo A. Altamura, Alessandra Bertoldo, Paolo Brambilla

**Affiliations:** 1 IRCCS “E. Medea” Scientific Institute, Bosisio Parini, Lecco, Italy; 2 Section of Psychiatry, AOUI Verona, Verona, Italy; 3 Section of Psychiatry, Department of Neurosciences, Biomedicine and Movement Sciences, University of Verona, Verona, Italy; 4 Section of Clinical Psychology, Department of Neurosciences, Biomedicine and Movement Sciences, Verona, Italy; 5 Department of Radiology, University of Verona, Verona, Italy; 6 Department of Neurosciences and Mental Health, Fondazione IRCCS Ca' Granda Ospedale Maggiore Policlinico, University of Milan, Milan, Italy; 7 Department of Information Engineering (DEI), University of Padova, Padova, Italy; 8 Department of Psychiatry and Behavioral Sciences, UTHouston Medical School, Houston, Texas, United States of America; National University of Defense Technology College of Mechatronic Engineering and Automation, CHINA

## Abstract

Several strands of evidence reported a significant overlapping, in terms of clinical symptoms, epidemiology and treatment response, between the two major psychotic disorders—Schizophrenia (SCZ) and Bipolar Disorder (BD). Nevertheless, the shared neurobiological correlates of these two disorders are far from conclusive. This study aims toward a better understanding of possible common microstructural brain alterations in SCZ and BD. Magnetic Resonance Diffusion data of 33 patients with BD, 19 with SCZ and 35 healthy controls were acquired. Diffusion indexes were calculated, then analyzed using Tract-Based Spatial Statistics (TBSS). We tested correlations with clinical and psychological variables. In both patient groups mean diffusion (MD), volume ratio (VR) and radial diffusivity (RD) showed a significant increase, while fractional anisotropy (FA) and mode (MO) decreased compared to the healthy group. Changes in diffusion were located, for both diseases, in the fronto-temporal and callosal networks. Finally, no significant differences were identified between patient groups, and a significant correlations between length of disease and FA and VR within the corpus callosum, corona radiata and thalamic radiation were observed in bipolar disorder. To our knowledge, this is the first study applying TBSS on all the DTI indexes at the same time in both patient groups showing that they share similar impairments in microstructural connectivity, with particular regards to fronto-temporal and callosal communication, which are likely to worsen over time. Such features may represent neural common underpinnings characterizing major psychoses and confirm the central role of white matter pathology in schizophrenia and bipolar disorder.

## Introduction

Several studies consistently reported that Bipolar Disorder (BD) and schizophrenia (SCZ) share clinical, cognitive and genetic aspects [1][2][3][4][5] overcoming the Kraepelinian dichotomy theory [6] which considers BD and SCZ as two distinct diseases. Indeed, in recent years, changes in diffusion measures in BD and SCZ have been investigated under the hypothesis that diffusion alterations, particularly in the white matter (WM), can unveil structural connectivity modifications characterizing major psychoses [7].

In this context, Diffusion Tensor Imaging (DTI) enables the investigation of microstructural alterations in the organization and orientation of WM tracts, through the extraction of specific diffusion indexes interconnected with each other [8][9]. Specifically, fractional anisotropy (FA) measures how much one direction is prevailing on the others inside a particular voxel, whereas mean diffusivity (MD) represents the magnitude of the diffusion. Axial (AD) and radial diffusivity (RD) measure the amount of diffusion that occurs along the principal axis of the diffusion tensor and perpendicularly to it. Volume ratio (VR) gives a measure of the ratio between the ellipsoid volume and that of a sphere having a radius equivalent to the mean diffusivity. Finally, mode (MO) [10], is related to the three-dimensional characteristics of anisotropy (from linear to planar). The analysis of these indexes provides insight into WM microstructural alterations.

Several studies observed similar alterations in cortical areas in SCZ and BD, within prefrontal, temporal and parietal lobes [11][12][13][14][15] and in subcortical regions, including the amygdala, the basal ganglia and the thalamus [16][17][18][14][19]. Furthermore, SCZ and BD have both been considered as “dysconnection syndromes” [20][21][22], being the symptoms sustained by abnormal regional interactions. However, SCZ has been investigated using DTI more widely than BD [12], both at region of interest (ROI) and at a voxel level. In particular, it has been reported that SCZ patients showed widespread lowered FA compared to healthy subjects in several brain regions, including frontal and parietal WM [11][12], internal capsule [23][24], temporal and occipital WM [11], corpus callosum [23][25][26], longitudinal fasciculus [27][28], anterior cingulate bundle [29] and uncinate fasciculus [27]. Additionally, changes were found also in terms of increased RD in the temporal gyrus [30] and increased MD in fronto-temporal areas [11], corpus callosum [31], amygdala [32], and thalamus [33].

With regard to BD, findings are not always in agreement, as suggested by previous studies from our group [34][35] and other independent studies [36][37]. Nonetheless, the majority of these studies detected a decrease in FA, which could be related to disruption of WM. Furthermore, similarly to SCZ, decreased FA was found in BD, both at ROI and at a voxel level, in various WM regions, including frontal WM tracts [38], corona radiata [39], anterior cingulum [40], corpus callosum and arcuate and uncinate fasciculi [36][41]. Although other DTI indexes are less investigated than FA, an increase in MD has been found in WM tracts [7][42] and in the frontal gyri [43], whereas increases in AD and RD have been reported in the fornix [44].

Finally, TBSS (Tract—Based Spatial Statistics [45]) studies also showed an involvement of all major WM tracts [46], located in the frontal, temporal [36][47][48], parietal and occipital WM areas [49] as well as corpus callosum and longitudinal fasciculus [50][51].

Despite the similarities in WM alterations in either BD or SCZ, these two psychiatric illnesses have rarely been studied together in terms of brain diffusion. In most cases no differences were found [13][37][49][52][53], except for a DTI study carried out by Lu and colleagues [54] reporting reduced FA in BD in selective ROIs, including the cingulum, internal capsule, posterior corpus callosum, posterior thalamic radiation and inferior longitudinal fasciculus/inferior fronto-occipital fasciculus WM compared to SCZ. Moreover, a recent TBSS study reported similar WM connectivity abnormalities in callosal, paralimbic and fronto-occipital regions in both SCZ and BD and also that dysconnectivity predicted functional outcome in both illnesses [49].

In this work we aim to delineate common and distinct WM abnormalities between BD and SCZ patients. Specifically, we directly compared BD, SCZ and healthy controls (HC) taking into account the whole set of DTI indexes simultaneously. This approach, differently from previous studies, where mostly FA and MD are analyzed, provides a comprehensive insight on the changes in diffusion characterizing BD and SCZ. Specifically, in respect to Kumar and colleagues [49], who also compared BD and SCZ with TBSS, we specifically aimed at finding regions where all diffusion indexes are compromised, instead of comparing other indexes were FA shows differences between healthy and patients, with the intent of exploiting all the information we can get with the DTI model.

To our knowledge, this is the first study applying TBSS on all the DTI indexes at the same time. Finally, the impact of age and clinical variables on diffusion values for both groups of patients was explored.

## Materials and methods

### Participants

Thirty-three patients with DSM-IV BD (18 type I, 15 type 2), 19 patients with SCZ and 35 HC were recruited. The recruitment of patients was made by means of the South-Verona Psychiatric Care Register (PCR) [55], a community-based mental health register. Diagnoses for SCZ and BD were obtained using the Item Group Checklist of the Schedule for Clinical Assessment in Neuropsychiatry (IGC-SCAN) (World Health Organization, 1992) and confirmed by the clinical consensus of two staff psychiatrists. The duration of illness was determined in years since symptoms onset. The clinical symptomatology was evaluated with the Brief Psychiatric Rating Scale (BPRS) in both disorders as well as with the Hamilton Depression Rating Scale (HDRS) and the Bech-Rafaelsen Mania Rating Scale (BRMRS) in patients with BD. All patients with other Axis I disorders, alcohol or substance abuse, history of traumatic head injury with loss of consciousness, epilepsy or other neurological or medical diseases, including hypertension and diabetes, were excluded from the study.

HC were recruited through word of mouth and advertisements in the local communities and had no history of head injury or psychiatric disorders, no psychiatric disorders among first-degree relatives, and no history of substance or alcohol abuse. The absence of psychiatric disorders was tested using an interview modified from the non-patient version of the SCID-IV. For all demographic and clinical details please refer to Table 1.

**Table 1:** Demographic and clinical details of subjects participating to the study.

		Healthy controls (N = 35)	Patients with Bipolar Disorder (N = 33,18 BD I, 15 BD II)	Patients with Schizophrenia (N = 19)
		Mean (SD)	Mean (SD)	Mean (SD)
Age (years, mean(SD))		39.0 (12.6)	48.9 (8.3)	46.1 (11.5)
Sex (males/females)*		19/16	8/25	13/6
Duration of Illness (years, mean(SD))		-	20.8 (10.5)	20.8 (12.6)
BPRS total score (mean(SD))		-	33.0 (8.3)	34.3 (6.7)
HDRS total score (mean(SD))		-	11.5 (11.8)	-
BRMRS total score (mean(SD))		-	3.1 (4.4)	-
Psychotic symptoms (yes/no)		-	20/13	19/0
Number of hospitalizations (mean(SD))		-	5.3 (4.8)	4.1 (4.9)
Medication	AP typical (chlorpromazine equivalents, yes/no, mean(SD)*	-	15/18 99.2 (104.1)	7/12 68.4 (41.6)
	AP atypical (chlorpromazine equivalents, yes/no, mean(SD)*	-	7/26 255.0 (142.0)	12/7 280.6 (123.8)
	AD (PDD/DDD, yes/no, mean(SD))	-	13/20 1.64 (0.63)	9/10 1.16 (0.65)
	BZD (PDD/DDD, yes/no, mean(SD)*	-	12/21 2.42 (3.84)	9/10 3.83 (4.87)
	STAB (PDD/DDD, yes/no, mean(SD)*	-	11/22 0.91 (0.24)	0/19

Demographic and clinical details of our sample. BD = bipolar disorder; SD = standard deviation; BPRS = Brief Psychiatric Rating Scale; HDRS = Hamilton Depression Rating Scale; BRMRS = Bech-Rafaelsen Mania Rating Scale; AP = Antipsychotics; AD = Antidepressants; BZD = benzodiazepines; STAB = Mood stabilizers.

*gender significantly different between samples (Chi-squared test, p < 0.05).

All the procedures were approved by the Biomedical Ethics Committee of the Azienda Ospedaliera di Verona and are in accordance with the Helsinki Declaration of 1975. All subjects signed a written informed consent to the protocol.

### MRI scanning

DTI images were acquired with a 1.5 T Siemens Symphony (Siemens Healthcare, Erlangen, Germany) scanner along 12 non-collinear directions with a single non-diffusion weighted image S_0_. The parameters used for the acquisition were as follows: repetition time (TR) = 8900 ms, echo time (TE) = 104 ms, matrix size 256 x 256x50, voxel size 0.92 x 0.92 x 3 mm^3^, b = 1000 s/mm^2^.

### Data analysis

Distortions due to eddy currents and small head motion were corrected aligning the diffusion-weighted images to the S_0_ using affine transformations utilizing the eddy currents correction tool from FSL [56][57]. The images were then skull-stripped using the brain extraction tool of FSL [58] for all the subjects. Subsequently, the data were fitted to the tensor model, as implemented in the function FDT in FSL [59][60], and FA, MD, RD, RA, and VR indexes were obtained. MO was calculated with an in-house MatLab script (MATLAB R2011b, The MathWorks Inc., Natick, MA, 2011) according to the work of Ennis and colleagues [10].

Patients were divided into two distinct groups according to the diagnosis. We compared age and gender between groups. We investigated differences in diffusion between patients affected by BD HC, and between patients affected by SCZ and HC. We also investigated if there were differences, in terms of diffusion, between BD I and BD II patients. Then, we compared SCZ with BD.

TBSS consists in various steps. Briefly, all FA images from all subjects were non-linearly registered to the FMBRIB-FA standard space using FNIRT [61] and a mean FA image was created. Then, the center of the WM tracts were delineated by thinning, to obtain a “skeleton” representing the brain WM tracts common to all subjects belonging to the group. The resulting data were processed applying voxel-wise cross-subject statistics, using FSL randomise function [62] (p<0.05). We corrected the results for multiple comparisons using threshold-free cluster enhancement (TFCE [63]), an approach similar to cluster-based thresholding. Age was treated as a nuisance variable to avoid spurious results related with the natural changes in diffusion associated with aging. Also gender was considered as a covariate of no interest. Moreover, mean values and standard deviations of the considered diffusion indexes in the affected WM tracts, identified using the JHU ICBM-DTI-81 (http://cmrm.med.jhmi.edu/) tract atlas, have been compared with the healthy population. We tested if diffusion indexes came from different statistical populations, using the Wilcoxon-Mann-Whitney rank-sum test (p = 0.05) for both comparisons.

We then investigated if there were areas or clusters of voxels where all diffusion indexes showed abnormal behavior simultaneously. To achieve this, the results obtained with the different indexes have been intersected, considering the voxels where the differences between healthy subjects and patients were statistically significant (p<0.05) for each index.

Finally, we investigated if diffusion indexes variations in the pathological population correlated with the duration of illness, use of medication, BPRS, HDRS and BRMRS score in the case of BD, and with length of disease, BPRS scores, medication and number of hospitalizations in SCZ subjects. Also in this case results were corrected for multiple comparisons considering only voxels with p < 0.05, identified using TFCE.

## Results

We found widespread white matter alterations both in BD and in SCZ. In particular, FA decreased, while MD, VR, AD and RD increased in both groups of patients in respect to healthy controls (TFCE, p < 0.05). In BD patients MO showed a decrease in small regions. The affected areas overlap substantially in BD and SCZ. In a direct comparison between BD and SCZ and between BD I and BD II no differences have been found in diffusion indexes (TFCE, p > 0.05). Gender differed between samples (Chi-squared test, p < 0.05), while age did not show a significant difference (Wilcoxon rank-sum test, p > 0.05).

### Direct comparisons of patients affected by SCZ and BD with HC

#### SCZ vs HC

For patients with SCZ, after multiple comparison correction (TFCE, p = 0.05) FA showed a decrease in respect to HC, while MD, VR, AD and RD showed an increase in widespread WM areas, including corpus callosum, corona radiata, longitudinal fasciculus, internal and external capsule, thalamic radiation (values in Table 2). Coherently, the Wilcoxon rank-sum test identified all indexes values to be different in all tracts (Table 2).

**Table 2:** Brain areas where diffusion indexes are different in schizophrenia patients in respect to healthy controls.

**FA**			
**Tract**	**SCZ patients**	**Healthy controls**	**rank-sum (p)**
	mean (SD) x10 ^2^	mean (SD) x10 ^2^	
Corpus callosum	0.70 (0.12)	0.73 (0.12)	<10^−3^
Corona radiata L	0.51 (0.10)	0.53 (0.099)	<10^−3^
Corona radiata R	0.51 (0.098)	0.53 (0.095)	<10^−3^
Longitudinal fasciculus L	0.51 (0.097)	0.53 (0.099)	<10^−3^
Longitudinal fasciculus R	0.51 (0.095)	0.53 (0.099)	<10^−3^
External capsule L	0.46 (0.11)	0.48 (0.11)	<10^−3^
External capsule R	0.47 (0.11)	0.48 (0.11	<10^−3^
Thalamic radiation L	0.62 (0.10)	0.65 (0.11)	<10^−3^
Thalamic radiation R	0.62 (0.10	0.65 (0.11)	<10^−3^
**MD**			
**Tract**	**SCZ patients**	**Healthy controls**	**rank-sum (p)**
	mean (SD) x10 ^4^	mean (SD) x10 ^4^	
Corpus callosum	8.17 (2.23)	7.63 (1.26)	<10^−3^
Corona radiata L	7.40 (0.94)	7.18 (0.85)	<10^−3^
Corona radiata R	7.43 (0.50)	7.17 (0.83)	<10^−3^
Internal capsule L	7.31 (0.81)	7.19 (0.77)	<10^−3^
Internal capsule R	7.37 (1.07)	7.22 (0.79)	<10^−3^
Longitudinal fasciculus L	7.25 (0.80)	7.02 (0.76)	<10^−3^
Longitudinal fasciculus R	7.29 (0.84)	7.05 (0.80)	<10^−3^
External capsule L	7.82 (0.83)	7.63 (0.76)	<10^−3^
External capsule R	7.77 (0.84)	7.64 (0.75)	<10^−3^
Thalamic radiation L	8.23 (.1.41)	7.72 (1.42)	<10^−3^
Thalamic radiation R	8.22 (2.69(	7.60 (1.30)	<10^−3^
**VR**			
**Tract**	**SCZ patients**	**Healthy controls**	**rank-sum (p)**
	mean (SD)	mean (SD)	
Corpus callosum	0.44 (0.19)	0.41 (0.19)	<10^−3^
Longitudinal fasciculus R L	0.70 (0.11)	0.67 (0.12)	<10^−3^
External capsule L	0.76 (0.12)	0.74 (0.12)	<10^−3^
External capsule R	0.75 (0.12)	0.74 (0.12)	<10^−3^
**AD**			
**Tract**	**SCZ patients**	**Healthy controls**	**rank-sum (p)**
	mean (SD) x10 ^3^	mean (SD)	
Corpus callosum	1.74 (0.48)	1.69 (0.45)	<10^−3^
Internal capsula R	1.35 (0.15)	1.34 (0.15)	<10^−3^
Corona radiata L	1.26 (0.21)	1.23 (0.17)	<10^−3^
Corona radiata R	1.27 (0.21)	1.23 (0.18)	<10^−3^
**RD**			
**Tract**	**SCZ patients**	**Healthy controls**	**rank-sum (p)**
	mean (SD) x10 ^4^	mean (SD) x10 ^4^	
Corpus callosum	3.96 (1.57)	3.57 (1.47)	<10^−3^
Corona radiata L	5.16 (1.03)	4.85 (0.95)	<10^−3^
Corona radiata R	5.15 (0.97)	4.83 (0.91)	<10^−3^
Internal capsule L	4.25 (0.94)	4.11 (0.93)	<10^−3^
Internal capsule R	4.24 (0.97)	4.12 (0.95)	<10^−3^
Longitudinal fasciculus L	5.03 (0.84)	4.76 (0.81)	<10^−3^
Longitudinal fasciculus R	5.05 (0.84)	4.79 (0.84)	<10^−3^
External capsule L	5.70 (1.06)	5.45 (1.02)	<10^−3^
External capsule R	5.65 (1.07)	5.45 (1.01)	<10^−3^
Thalamic radiation L	4.91 (1.35)	4.42 (1.44)	<10^−3^
Thalamic radiation R	4.59 (1.32)	4.24 (1.32)	<10^−3^

Brain areas where diffusion indexes are statistically different between patients affected by schizophrenia and healthy controls, mean and standard deviations of their values (FA = fractional anisotropy, MD = mean diffusivity, VR volume ratio, RD = radial diffusivity, L = left, R = right, SD = standard deviation) and results of the Wilcoxon rank-sum test.

**Fig 1:**
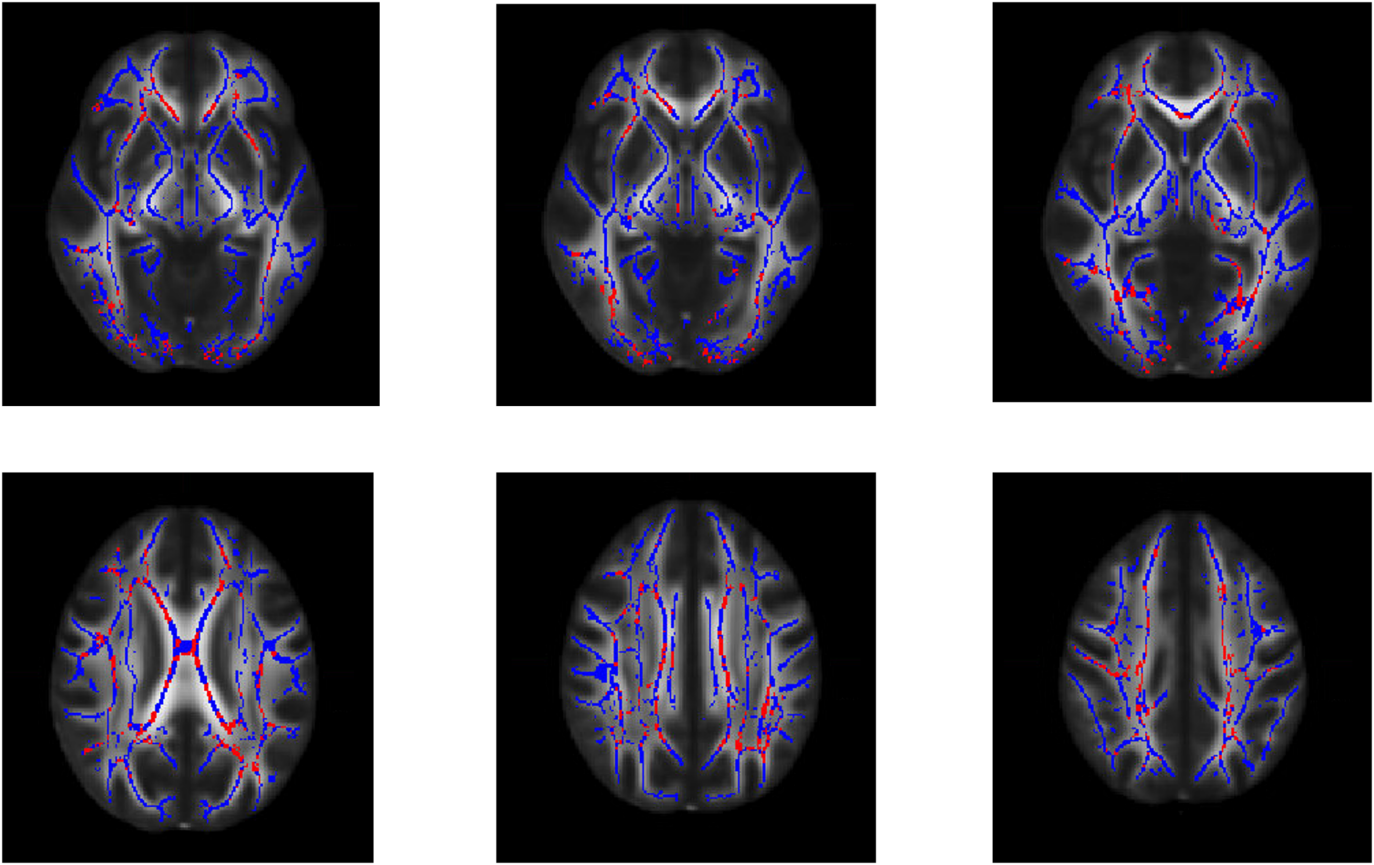
Areas with multiple altered DTI indexes in schizophrenia. White Matter tracts areas where fractional anisotropy, mean diffusivity, axial diffusivity, volume ratio and radial diffusivity show all a significant change in patients affected by schizophrenia, in respect to healthy individual (in red, p = 0.05, corrected with threshold-free cluster enhancement). Areas where all the considered indexes differ from values of healthy controls are corpus callosum, corona radiata, superior longitudinal fasciculus. The white matter skeleton is depicted in blue.

Areas where FA, MD, VR, AD and RD differ from HC are corpus callosum, right anterior thalamic radiation, left posterior thalamic radiation, superior and posterior corona radiata, longitudinal fasciculus (Fig 1).

#### BD vs HC

In BD patients FA, MD, VR, AD, MO and RD showed, after correction for multiple comparisons (TFCE, p = 0.05), significant differences (mean values in Table 3) in many areas, including bilateral internal capsule and corpus callosum, bilateral external capsule, corona radiata, internal capsule and longitudinal fasciculus. Additionally, MO decreased only in small areas located in the corpus callosum and in the internal capsule.

**Table 3:** Brain areas where diffusion indexes are different in bipolar disorder patients in respect to healthy controls.

**FA**			
**Tract**	**BD patients**	**Healthy controls**	**rank-sum (p)**
	mean (SD) x10 ^2^	mean (SD) x10 ^2^	
Corpus callosum	0.69 (0.13)	0.73 (0.12)	<10^−3^
Corona radiata L	0.49 (0.097)	0.53 (0.099)	<10^−3^
Corona radiata R	0.50 (0.098)	0.53 (0.095)	<10^−3^
Internal capsule L	0.63 (0.095)	0.65 (0.094)	<10^−3^
Internal capsule R	0.63 (0.093)	0.65 (0.095)	<10^−3^
Longitudinal fasciculus L	0.51 (0.098)	0.53 (0.099)	<10^−3^
Longitudinal fasciculus R	0.51 (0.099)	0.53 (0.099)	<10^−3^
**MD**			
**Tract**	**BD patients**	**Healthy controls**	**rank-sum (p)**
	mean (SD) x10 ^4^	mean (SD) x10 ^4^	
Corpus callosum	8.13 (1.51)	7.63 (1.26)	<10^−3^
Corona radiata L	7.50 (0.93)	7.18 (0.85)	<10^−3^
Corona radiata R	7.49 (0.92)	7.17 (0.83)	<10^−3^
Internal capsule L	7.39 (0.85)	7.19 (0.77)	<10^−3^
Internal capsule R	7.43 (0.83)	7.22 (0.79)	<10^−3^
Longitudinal fasciculus L	7.24 (0.76)	7.02 (0.76)	<10^−3^
Longitudinal fasciculus R	7.29 (0.78)	7.05 (0.80)	<10^−3^
External capsule L	7.84 (0.81)	7.63 (0.76)	<10^−3^
External capsule R	7.74 (0.79)	7.64 (0.75)	<10^−3^
Thalamic radiation L	8.18 (.1.37)	7.72 (1.42)	<10^−3^
Thalamic radiation R	8.21 (2.57)	7.60 (1.30)	<10^−3^
**VR**			
**Tract**	**BD patients**	**Healthy controls**	**rank-sum (p)**
	mean (SD)	mean (SD)	
Corpus callosum	0.47 (0.20)	0.41 (0.19)	<10^−3^
Corona radiata L	0.73 (0.11)	0.68 (0.12) 68	<10^−3^
Corona radiata R	0.72 (0.11)	0.68 (0.12)	<10^−3^
Longitudinal fasciculus R L	0.70 (0.12)	0.68 (0.12)	<10^−3^
External capsule L	0.77 (0.12)	0.74 (0.12)	<10^−3^
External capsule R	0.76 (0.12)	0.73 (0.12)	<10^−3^
Cingulum L	0.65 (0.13)	0.59 (0.15)	<10^−3^
**AD**			
**Tract**	**BD patients**	**Healthy controls**	**rank-sum (p)**
	mean (SD) x10 ^3^	mean (SD) x10 ^3^	
Corona radiata R	1.20 (0.16)	1.19 (0.15)	<10^−3^
Longitudinal fasciculus R	1.18 (0.17)	1.16 (0.18)	<10^−3^
Internal capsule R	1.35 (0.16)	1.34 (0.15)	<10^−3^
**RD**			
**Tract**	**BD patients**	**Healthy controls**	**rank-sum (p)**
	mean (SD) x10 ^4^	mean (SD) x10 ^4^	
Corpus callosum	4.21 (1.73)	3.57 (1.47)	<10^−3^
Corona radiata L	5.30 (1.01)	4.85 (0.95)	<10^−3^
Corona radiata R	5.25 (1.00)	4.83 (0.91)	<10^−3^
Internal capsule L	4.35 (0.99)	4.11 (0.93)	<10^−3^
Internal capsule R	4.38 (0.97)	4.13 (0.95)	<10^−3^
Longitudinal fasciculus L	5.05 (0.84)	4.76 (0.81)	<10^−3^
Longitudinal fasciculus R	5.05 (0.87)	4.79 (0.84)	<10^−3^
**MO**			
**Tract**	**BD patients**	**Healthy controls**	**rank-sum (p)**
	mean (SD)	mean (SD)	
Corpus callosum	0.86 (0.18)	0.88 (0.16)	<10^−3^

Brain areas where diffusion indexes are statistically different between patients affected by bipolar disorder and healthy controls, mean and standard deviations of their values ((FA = fractional anisotropy, MD = mean diffusivity, VR volume ratio, RD = radial diffusivity, MO = mode, L = left, R = right, SD = standard deviation) and results of the Wilcoxon rank-sum test.

**Fig 2:**
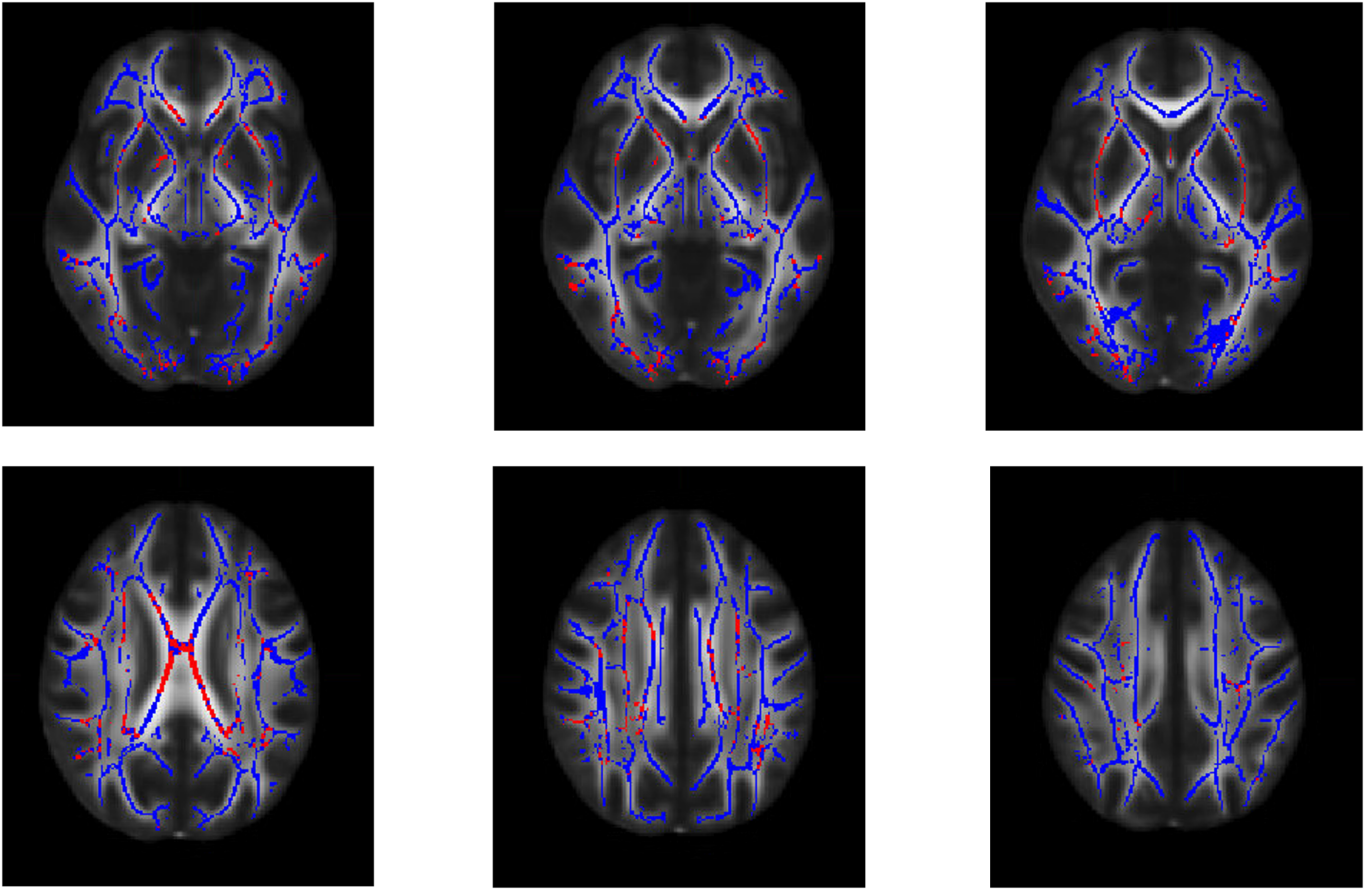
Areas with multiple altered DTI indexes in bipolar disorder. White matter tracts areas where fractional anisotropy, mean diffusivity, volume ratio, axial diffusivity and radial diffusivity show all a significant change in respect to healthy individual (in red, p = 0.05, corrected with threshold-free cluster enhancement) in patients with bipolar disorder. It can be noted that clusters of voxels, located in particular in the corpus callosum, in the external capsule and internal capsule show abnormalities in all the considered diffusion indexes. The white matter skeleton is depicted in blue.

Due to the small amount of voxels where MO was found to be sensitive to pathology, we did not consider MO in the computation of the areas which showed a variation of all indexes simultaneously. FA, MD, VR, AD and RD showed significant changes in respect to HC in particular in the corpus callosum, external capsule and internal capsule.(Fig 2).

#### BD vs SCZ

The direct comparison between the two groups of patients, after correction for multiple comparisons, did not show statistically relevant differences. Also, no differences in diffusion indexes were found between BD I and BD II patients.

### 2. Correlations of diffusion values of patients affected by SCZ and BD with clinical indexes

In patients affected by SCZ, we did not find any statistically significant correlation with clinical variables after multiple comparison correction (TFCE, p = 0.05).

In patients affected by BD, length of illness showed statistically significant negative correlations with FA in internal and external capsule and corona radiata as well as positive correlations with VR in internal and external capsule, corona radiata, corpus callosum (S1 Table, S1 and S2 Figs).

## Discussion

In this work, we have taken into account the whole set of DTI indexes (FA, MD, VR, RD, AD and MO), with the aim of reaching a comprehensive understanding of brain diffusion in BD and SCZ. Diffusion resulted to be affected both in BD and in SCZ patients, whereas no significant differences were found between the two groups of patients. In particular, patients suffering from BD and SCZ showed widespread reduction of FA and a significant increase in all the other indexes in all major WM connections compared to healthy subjects. Only for patients affected by SCZ, AD did not show significant differences when compared with HC.

These results suggest that the alterations underlying the pathophysiology of SCZ and BD are similar when structural connectivity, in terms of diffusion of water in the brain, is considered. Specifically, in this study, BD and SCZ patients showed similar dysfunctional patterns in MD and FA values, in respect to HC, further suggesting that structural connectivity is similarly affected in these two diseases, in line with previous findings [7][11][27][30][49][64].

It is to be noted that more than half BD patients who took part to this study have a lifetime history of psychosis. Psychotic symptoms have recently been seen as representing an intermediate phenotype between bipolar disorder with no psychotic symptoms and schizophrenia [65; 66; 67]: this could partly explain our results. Interestingly, as can be seen in Table 1, medication intake is similar in the two patient groups, thus this should not add any major bias to our findings.

Indeed, FA and MD have been found to decrease in both groups of patients in many brain areas, especially in prefrontal cortex (PFC), corpus callosum, corona radiata, longitudinal fasciculi and internal capsule [11][49]. Moreover, a relationship between the alteration of those tracts and specific cognitive profiles has been largely reported in both disorders [39][68][69][70][71]. In particular, Karbasforoushan and colleagues [68] found that decreases in the integrity of corpus callosum, cingulum, superior and inferior frontal gyri, and precuneus, are related with processing speed impairment, whereas Bauer and colleagues [39] showed that BD patients with alterations of FA, RD and MD within the internal capsule, the superior and anterior corona radiata, and the corpus callosum had low performances on verbal fluency tasks.

Similarly, emotional and cognitive processes involved in SCZ have been linked to modifications in the internal capsule and its fronto-thalamic network [24][37][72], potentially supporting dysfunctional connectivity and altered executive functions [69].

Moreover, a decrease in FA, coupled with the increase in MD, RD and VR, which has been found in both SCZ and BD in our sample, could be the consequence of disrupted brain connections and/or demyelination [9]. Increases in AD, VR, MD, observed in BD, might indicate that the structure of tissues is damaged, with a consequent impact on the boundaries needed for the diffusion. These findings are in line with a previous work from our research network [50], which reported a decrease in FA and an increase in MD and AD in an independent group of patients with BD. Furthermore, a decrease in FA coupled with an increase in RD observed in corpus callosum, thalamic radiation, corona radiata in SCZ, and in widespread WM areas in BD, could indicate that most damage has been done to myelin sheaths and barriers perpendicular to the main axis of the axons [9]. Finally, an alteration also in AD, as can be seen in our group of BD patients, indicates that damage occurs also along the main direction of diffusion, i.e. along the axons and neural pathways.

Our results also showed a significant WM impairment in corpus callosum in both diseases, which our group already demonstrated in terms of volume reduction and signal intensity in BD [73][74]. These findings confirm that inter-hemispheric connectivity is hindered in both the disorders [7], representing a common neural underpinning of major psychoses [35][75][76]. The corpus callosum is known to play a fundamental role by modulating inter-hemispheric communication and cognitive processes [77][78]. In particular, the anterior callosal fibers connect the bilateral frontal cortices including the cortices associated with several cognitive domains such as memory, attention, and executive functions [78]. The integrity of the corpus callosum is therefore crucial for sustained attention, context processing and language, which are frequently impaired in BD and SCZ [68][77][79].

Interestingly, we found AD to be higher in BD patients in respect to healthy controls, while no significant differences were found for SCZ patients. These changes, in BD patients, are located particularly in the right fronto-temporal network, in particular in corona radiata, superior longitudinal fasciculus and internal capsule. AD is often associated with axonal integrity [73][80] and it has been previously found increased in both BD and SCZ [30][44][54]. This suggests, especially when coupled with an increase in RD, that water content is augmented outside the axons, mirroring a loss of WM integrity [9]. The fact that we found no evidence in changes in AD for patients with SCZ is also supported by the literature [81][82] and reveals that damage to axons is more evident in BD than in SCZ. In contrast, both diseases are characterized by damage to myelin, proved by increases in diffusion perpendicularly to the main axis of axons, i.e. increases in RD [83].

Our results did not show any significant correlation between psychopathological measures and diffusion in WM tracts, similarly to previous findings [84][85]. However, we found clusters of voxels where the length of disease correlated with FA and VR in bipolar disorder, suggesting that the damage to WM progresses with chronicity. Specifically, the areas where diffusion disruption was related to length of illness were located in the corpus callosum, in external and internal capsule, corona radiata and thalamic radiation. All together, these results suggests that there is a noticeable impairment of inter-hemispheric and fronto-temporal connectivity in BD, particularly worsening with chronicity. Our results may in part be associated to the long duration of illness of our sample, which could have contributed to the changes detected in brain tissue integrity. It is to be noted that in our SCZ sample the correlations were significant before multiple comparison correction, and did not survive the correction, probably due to small sample size. In order to confirm our speculation, longitudinal DTI studies would be needed.

Our findings suffer from two major limitations, which may limit the generalizability of the results. First, the sample size was relatively small and composed by chronic patients, although it was comparable with most of the available recent studies (e.g. [86][87]). Second, this study was conducted with a relatively low resolution DTI acquisition, which, even if it does not affect any of the considerations about water diffusion, could partially detect fine tissue details. Moreover, our dataset was composed of subjects who had a long story of illness that could partially interfere with the results and cause non-specific WM damage. Finally the age at onset of our sample was higher than generally expected, even if similar in the two patient groups.

In conclusion, this study shows, through an extensive analysis of DTI indexes, that BD and SCZ share similar impairments in microstructural connectivity, particularly in fronto-temporal and callosal communication, which also seem to be affected by the progression of the illnesses. Such features may represent neural common underpinnings characterizing major psychoses and confirm the central role of WM pathology in SCZ and BD, adding further evidence for a dimensional continuum of these two disorders in the light of searching for neural biomarkers across the psychosis spectrum [88]. In this perspective, future larger longitudinal studies should explore both structural and functional connectivity in first episode psychotic patients to further characterize the etiology of the two diseases.

## Supporting information

S1 Fig Correlation of fractional anisotropy with length of disease in bipolar disorder. Areas where fractional anisotropy values significantly negatively correlate (in red, p = 0.05, corrected with threshold-free cluster enhancement) with length of disease in patients with bipolar disorder. Some clusters can be identified, located in particular in external capsule and corona radiata. The white matter skeleton is depicted in blue.
(TIF)

S2 Fig Correlation of volume ratio with length of disease in bipolar disorder. Areas where volume ratio values significantly positively correlate (p = 0.05, corrected with threshold-free cluster enhancement) with length of disease in patients with bipolar disorder. Some clusters can be identified, located in particular in external capsule, corona radiata, corpus callosum and internal capsule.
(TIF)

S1 Table White matter structures and DTI indexes which correlate with length of disease in bipolar disorder. List of white matter structures and DTI indexes which show a significant correlation with length of disease (p = 0.05, corrected with threshold-free cluster enhancement) in patients affected by bipolar disorder. Indexes which resulted significant in this analysis are fractional anisotropy (FA) and volume ratio (VR) (L = left, R = right).
(DOCX)

S1 File List of subjects involved in the study: diagnosis, age, gender and length of disease. CNT = healthy control; BD = bipolar disorder; SCZ = schizophrenia; F = female; M = male.
(XLSX)
