# Supplementary figures and images for: Similar white matter changes in schizophrenia and bipolar disorder: A tract-based spatial statistics study
Source: PLoS One. 2017 Jun 28;12(6):e0178089. doi: 10.1371/journal.pone.0178089 (PMC5489157; doi:10.1371/journal.pone.0178089)

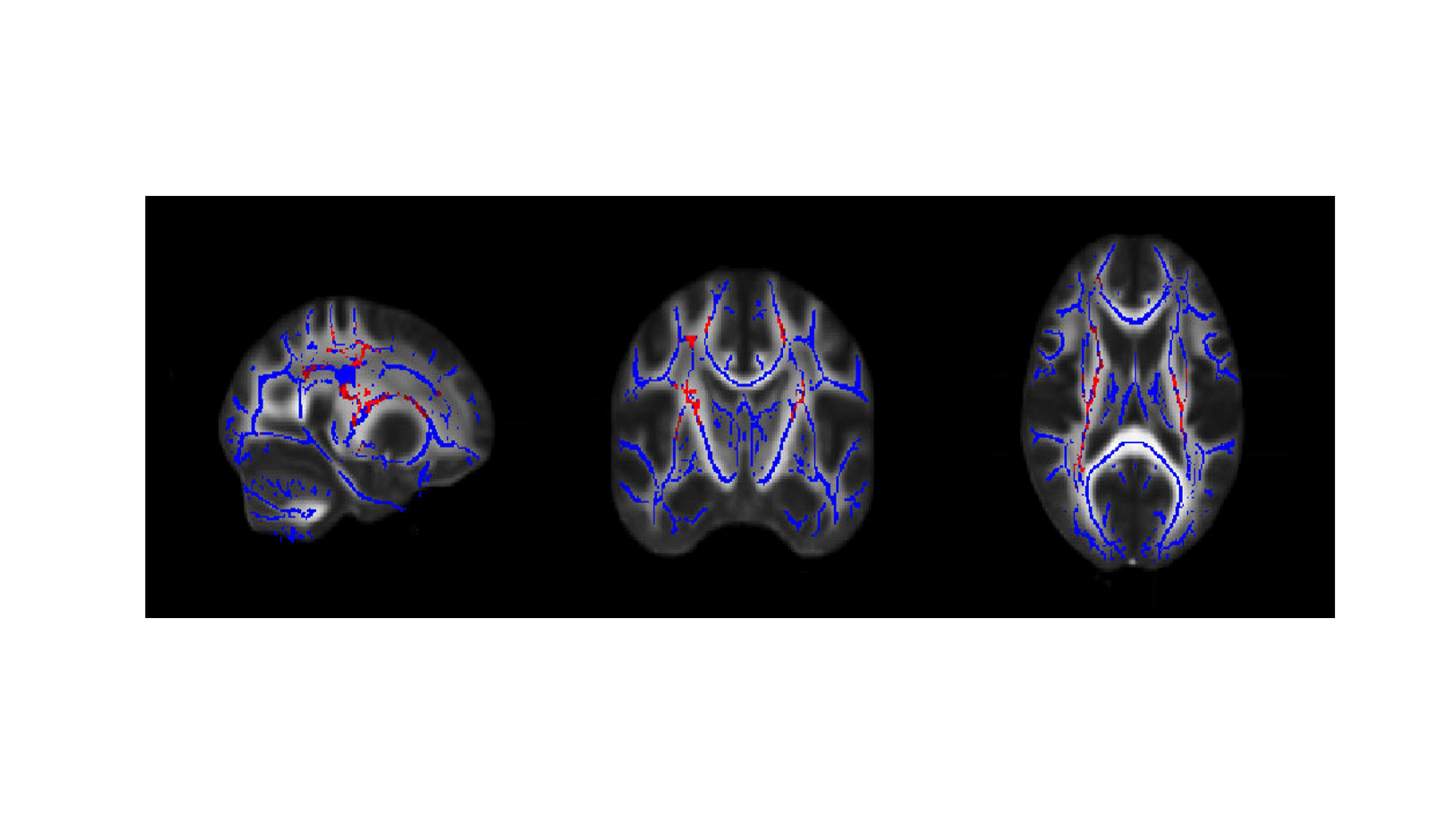

Supplement: S1 Fig — Areas where fractional anisotropy values significantly negatively correlate (in red, p = 0.05, corrected with threshold-free cluster enhancement) with length of disease in patients with bipolar disorder. Some clusters can be identified, located in particular in external capsule and corona radiata. The white matter skeleton is depicted in blue. (TIF) [file pone.0178089.s001.tif]

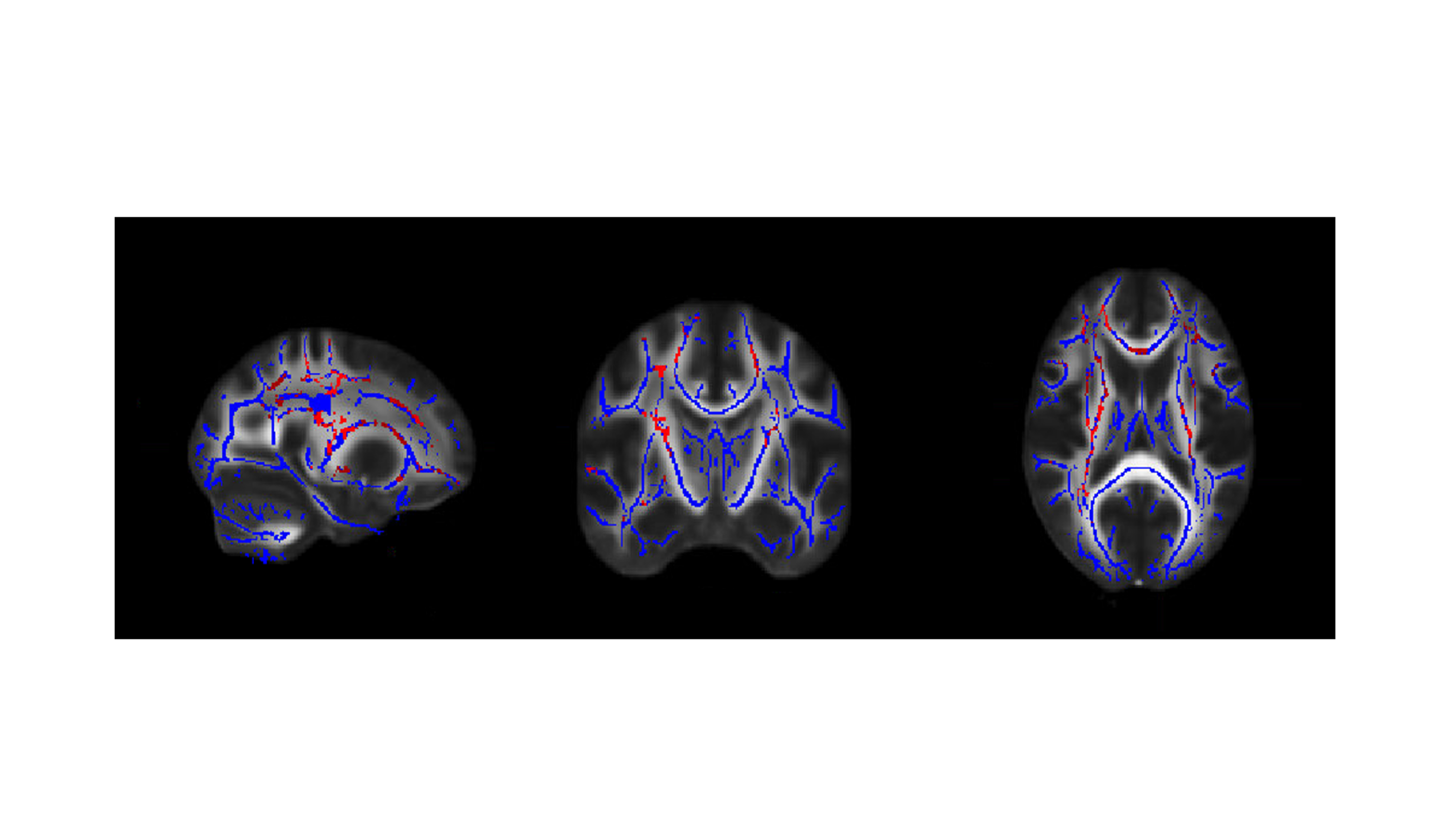

Supplement: S2 Fig — Areas where volume ratio values significantly positively correlate (p = 0.05, corrected with threshold-free cluster enhancement) with length of disease in patients with bipolar disorder. Some clusters can be identified, located in particular in external capsule, corona radiata, corpus callosum and internal capsule. (TIF) [file pone.0178089.s002.tif]
