# Supplementary material for: Similar white matter changes in schizophrenia and bipolar disorder: A tract-based spatial statistics study
Source: PLoS One. 2017 Jun 28;12(6):e0178089. doi: 10.1371/journal.pone.0178089 (PMC5489157; doi:10.1371/journal.pone.0178089)
Supplement: S1 Table — List of white matter structures and DTI indexes which show a significant correlation with length of disease (p = 0.05, corrected with threshold-free cluster enhancement) in patients affected by bipolar disorder. Indexes which resulted significant in this analysis are fractional anisotropy (FA) and volume ratio (VR) (L = left, R = right). (DOCX) [file pone.0178089.s003.docx]

**S1 Table**

| **DTI index** | **Structures** | **Relation** |
| --- | --- | --- |
| **FA** | L and R superior corona radiata  L and R anterior corona radiata  L and R interior capsule  L and R exterior capsule | Negative correlation |
| **VR** | R external capsule  L and R anterior corona radiata  L and R superior corona radiata  L and R posterior corona radiata  Corpus callosum: genu | Positive correlation |
